# Substitute sweeteners: diverse bacterial oligosaccharyltransferases with unique *N*-glycosylation site preferences

**DOI:** 10.1038/srep15237

**Published:** 2015-10-20

**Authors:** Anne A. Ollis, Yi Chai, Aravind Natarajan, Emily Perregaux, Thapakorn Jaroentomeechai, Cassandra Guarino, Jessica Smith, Sheng Zhang, Matthew P. DeLisa

**Affiliations:** 1 School of Chemical and Biomolecular Engineering, Cornell University, Ithaca, NY 14853 USA; 2 Department of Microbiology, Cornell University, Ithaca, NY 14853 USA; 3 Comparative Biomedical Sciences, Cornell University, Ithaca, NY 14853 USA; 4 Proteomics and Mass Spectrometry Core Facility, Cornell University, Ithaca, New York 14853

## Abstract

The central enzyme in the *Campylobacter jejuni* asparagine-linked glycosylation pathway is the oligosaccharyltransferase (OST), PglB, which transfers preassembled glycans to specific asparagine residues in target proteins. While *C. jejuni* PglB (*Cj*PglB) can transfer many diverse glycan structures, the acceptor sites that it recognizes are restricted predominantly to those having a negatively charged residue in the −2 position relative to the asparagine. Here, we investigated the acceptor-site preferences for 23 homologs with natural sequence variation compared to *Cj*PglB. Using an ectopic trans-complementation assay for *Cj*PglB function in glycosylation-competent *Escherichia coli*, we demonstrated *in vivo* activity for 16 of the candidate OSTs. Interestingly, the OSTs from *Campylobacter coli, Campylobacter upsaliensis*, *Desulfovibrio desulfuricans*, *Desulfovibrio gigas*, and *Desulfovibrio vulgaris*, exhibited significantly relaxed specificity towards the −2 position compared to *Cj*PglB. These enzymes glycosylated minimal N-X-T motifs in multiple targets and each followed unique, as yet unknown, rules governing acceptor-site preferences. One notable example is *D. gigas* PglB, which was the only bacterial OST to glycosylate the Fc domain of human immunoglobulin G at its native ‘QYNST’ sequon. Overall, we find that a subset of bacterial OSTs follow their own rules for acceptor-site specificity, thereby expanding the glycoengineering toolbox with previously unavailable biocatalytic diversity.

The covalent modification of protein side chains with glycans, protein glycosylation, is one of the most prolific post-translational modifications found in eukaryotes. Accordingly, protein glycosylation has important roles in numerous biological pathways and processes^1^. The most common type of protein glycosylation is asparagine-linked (*N*-linked) glycosylation, where modification occurs to the amide nitrogen of asparagine residues located within a glycosylation motif, or sequon, defined as N-X-S/T, where X is any residue but proline^2^. While initially thought to be exclusive to eukaryotes, *N-*linked glycosylation is now known to occur in all domains of life^3^.

Eukaryotic and prokaryotic *N*-linked glycosylation systems share many mechanistic features^4^. Both involve enzymatic synthesis of a lipid-linked oligosaccharide (LLO) donor and transfer of the preassembled glycan from the lipid to the sequon of a target protein in a reaction that is catalyzed by an oligosaccharyltransferase (OST). Some notable differences have also been observed. For example, the oligosaccharides attached in these systems are chemically distinct and the OSTs that catalyze their transfer are also distinct. In higher eukaryotes, the OST is comprised of eight different membrane proteins of which the catalytic subunit is STT3^5^, whereas in kinetoplastids and prokaryotes the OST is a monomeric enzyme bearing homology to STT3^6^. A further distinguishing feature is the observation that the prototypical bacterial OST, *Campylobacter jejuni* PglB (*Cj*PglB), exhibits broad selectivity for the glycan donor, permitting the transfer of structurally diverse oligosaccharides^7^,^8^ in addition to its native heptasaccharide GalNAc-α1,4-GalNAc-α1,4-(Glcβ1,3)-GalNAc-α1,4-GalNAc-α1,4-GalNAc-α1,3-Bac [where Bac is bacillosamine or 2,4-diacetamido−2,4,6-trideoxyglucose]^9^. However, while selectivity toward the glycan donor is relaxed, *Cj*PglB recognizes a more stringent protein acceptor site compared to the N-X-S/T (X ≠ P) sequon recognized by eukaryotic OSTs. Specifically, *Cj*PglB requires an acidic residue in the −2 position of the sequon, D/E-X_−1_-N-X_+1_-S/T (X_−1, +1_ ≠ P)^10^, with DQNAT having been defined experimentally as the optimal acceptor sequence^11^. In stark contrast, amino acids with an acidic side chain are disfavored at the −2 position in eukaryotic glycosylation sites^12^.

The “minus two rule”^13^ governing acceptor-site specificity of *Cj*PglB is considered an obstacle for efficient glycosylation of eukaryotic targets by bacterial OSTs^14^. To eliminate this potential bottleneck, we recently developed a high-throughput screening method named glycoSNAP, which was subsequently used to engineer *Cj*PglB variants that break this rule^15^. To date, however, very few studies have investigated the extent to which this rule may restrict other naturally occurring OSTs beyond *Cj*PglB. Two notable exceptions were reported recently whereby homologous PglBs from *Campylobacter lari* (*Cl*PglB) and *Desulfovibrio desulfuricans* (*Dd*PglB) were evaluated using reconstituted protein glycosylation pathways in *Escherichia coli* cells^16^. Interestingly, both *Cl*PglB and *Dd*PglB catalyzed a low level of glycosylation at a nonconsensus sequon, NNN_274_ST, in AcrA^13^,^17^, a naturally occurring *N-*linked glycoprotein from *C. jejuni* that serves as a model glycosylation target. This same site in AcrA was not recognized by *Cj*PglB^13^,^17^. However, the relaxed glycosylation was only observed for this unique site in AcrA and not with any other N-X-S/T sites tested including the native ‘QYNST’ acceptor site in the Fc domain of human immunoglobulin G^17^, suggesting that this non-canonical activity was not a broadly defining characteristic but rather a minor exception to the *Cj*PglB minus two rule. Interestingly, whereas glycosylation by *Cl*PglB was still most efficient when an acidic residue was present in the −2 position^17^, *Dd*PglB did not detectably glycosylate the canonical D/E-X_−1_-N-X_+1_-S/T sites in AcrA^13^. This latter observation suggests that the acceptor site preferences of *Dd*PglB are different from *Cj*PglB. While the glycosylation sites preferred by *Dd*PglB remain undetermined, the identification of an *N-*linked glycan in the crystal structure of the periplasmic 16 heme cytochrome HmcA from the closely related bacterium *Desulfovibrio gigas* may provide a clue. Specifically, a glycan comprised of three *N*-acetylhexosamine (HexNAc) molecules was covalently bound to N261 of HmcA in the motif _259_TANGT_263_^18^, which lacks a negatively charged amino acid in the −2 position. This would suggest that *Dg*PglB may not follow the minus two rule seen for *Cj*PglB; however, the substrate specificity of *Dg*PglB remains to be unequivocally determined.

To better understand the rules defining acceptor-site specificity for bacterial OSTs beyond *Cj*PglB, we systematically characterized the activity of a collection of PglB homologs from diverse Gram-negative bacterial species. The genomes of at least 49 bacterial species encode genes with homology to *Cj*PglB^19^. These putative OSTs are most commonly found in epsilonproteobacteria with a much smaller number identified in the genomes of deltaproteobacteria and aquificae. Here, a total of twenty-four candidate OSTs were functionally screened using an ectopic trans-complementation strategy in *E. coli* cells that carried the 17-kb *C. jejuni* protein glycosylation (*pgl*) locus with the *pglB* gene insertionally inactivated. While the majority of active OSTs preferred a negatively charged residue in the −2 position, akin to *Cj*PglB, we identified five OSTs that recognized a broader range of acceptor sites. Among these, *D. gigas* PglB (*Dg*PglB) exhibited the most relaxed specificity based on its unique ability to tolerate nearly any residue in the −2 position and glycosylate the Fc domain of human immunoglobulin G (IgG), the first example of native Fc glycosylation in *E*. *coli*.

## Results

### Mining of bacterial genomes yields diverse bacterial OSTs

**Figure 1:**
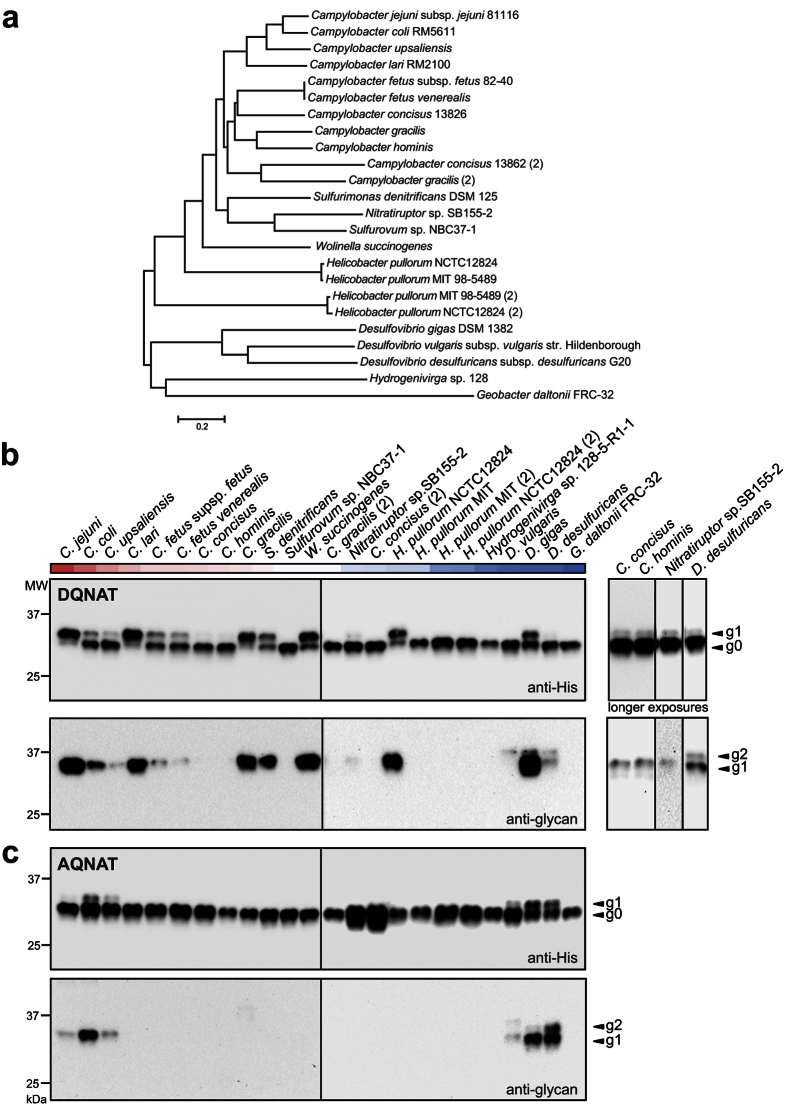
Select PglB homologs exhibit relaxed sequon specificity. (**a**) Phylogenetic tree of the PglB homologs examined in this study. The tree was constructed using Mega 6 software and the neighbor-joining method from a multiple sequence alignment. Species with two copies of PglB are denoted by (2) next to the copy with lowest sequence identity to *C*. *jejuni* PglB. (**b**,**c**) Immunoblot analysis of TCA-precipitated periplasmic fractions derived from CLM24 cells complemented with one of the PglB homologs indicated, and co-expressing the biosynthetic pathway for the *C*. *jejuni* heptasaccharide and either scFv13-R4^DQNAT^ (**b**) or scFv13-R4^AQNAT^ (**c**). OSTs are arranged in order of decreasing sequence identity to *C*. *jejuni* PglB based on a Clustal omega multiple sequence alignment percent identity matrix and indicated by the red (closely related) to blue (distantly related) heatmap across the top. Blots were probed with anti-His antibodies against the C-terminal polyhistidine tag on acceptor protein or anti-glycan hR6 serum reactive with the *C*. *jejuni* heptasaccharide. The g0 and g1 arrows indicate un- and monoglycosylated acceptor proteins, respectively. Molecular weight (MW) markers are indicated on the left. The panel on the right shows longer exposures, cropped from the same immunoblots on the left, to reveal activity for inefficiently glycosylated targets.

To gain a comprehensive view of potential OSTs, we assembled a collection of 24 candidate OSTs (from 18 sequenced bacterial genomes) with similarity to *Cj*PglB. This panel spanned four classes of bacteria, with 19 OSTs from species belonging to the epsilonproteobacteria (including *C. jejuni*, closely related *C. lari*, *Campylobacter coli*, and *Campylobacter upsaliensis*, and more distantly related *Helicobacter pullorum*, *Nitratiruptor* sp., *Sulfurimonas denitrificans*, and *Wolinella succinogenes*), 4 OSTs from deltaproteobacteria (*Geobacter* and *Desulfovibrio* sp.), and 1 OST from aquificae (*Hydrogenivirga* sp.) (Fig. 1a). The PglB homologs from *C. lari*, *C. upsaliensis*, and *C. coli* share 56, 65, and 81% sequence identity with *Cj*PglB, respectively, while those from the more distantly related epsilonproteobacteria share less sequence identity, ranging from 24–50%. The deltaproteobacteria and aquificae share the lowest sequence identity with *Cj*PglB, at 15–20%. Five epsilonproteobacteria, including *C. concisus*, *C. gracilis*, and 2 subspecies of *H. pullorum*, possess two PglB homologs (PglB1 and PglB2) that are located in different regions of the bacterial genome and share 25–38% sequence identity between pairs^19^,^20^.

### Identification of 15 PglB homologs that recognize the DQNAT sequon

To determine which of the identified OSTs could glycosylate the canonical DQNAT motif, we employed an ectopic trans-complementation strategy. This involved functionally transferring the *C. jejuni pgl* locus into *E. coli* K12, which lacks native *N*-linked glycosylation^16^. The recipient *E. coli* cells gain the ability to recombinantly produce acceptor proteins modified with the *C*. *jejuni* heptasaccharide GalNAc_5_(Glc)Bac. By insertionally inactivating the *pglB* gene in this plasmid, candidate PglB homologs can be provided *in trans* and readily tested for their ability to restore glycosylation activity in *E. coli*^13^,^15^,^20^. This strategy takes advantage of the fact that the core donor glycan structure from *C*. *jejuni* is conserved in the epsilonproteobacteria^21^,^22^ and that the distantly related *Dd*PglB can transfer the *C. jejuni* heptasaccharide^13^.

Genes coding for each of the OSTs were independently cloned into plasmid pSF, and the resulting plasmids were transformed into *E. coli* strain CLM24 carrying plasmid pACYC*pglB*::*kan*, which encodes the entire *C. jejuni pgl* locus but with the gene encoding *Cj*PglB inactivated by insertion of a kanamycin resistance cassette^23^. These cells were transformed with a third plasmid, pBS-scFv13-R4^DQNAT^, that encodes a single-chain Fv (scFv) antibody fragment fused with an N-terminal co-translational Sec export signal and a C-terminal DQNAT glycosylation tag^8^. We chose scFv13-R4^DQNAT^ because it is well expressed in the *E. coli* periplasm and can be efficiently glycosylated by *Cj*PglB^8^,^15^. The glycosylation status of the periplasmic scFv13-R4^DQNAT^ was analyzed by SDS-PAGE and immunoblotting with polyhistidine epitope tag-specific (anti-His) antibodies or *C*. *jejuni* heptasaccharide glycan-specific antiserum hR6^17^. Control cells complemented with *Cj*PglB produced two proteins reactive towards the anti-His antibodies, which corresponded to the un- and monoglycosylated forms of scFv13-R4^DQNAT^ as confirmed by their reactivity towards the hR6 antiserum (Fig. 1b). Likewise, 15 of the PglB homologs complemented the loss of the *Cj*PglB enzyme under the conditions tested here (Fig. 1b). This is the first described activity for 12 of the candidate OSTs, whereas activity for *Cl*PglB, *H. pullorum* PglB (*Hp*PglB) , and *Dd*PglB was previously demonstrated^6^,^13^,^17^,^20^. This is also the first example of consensus-site glycosylation by *Dd*PglB, as previous studies found that this enzyme did not detectably glycosylate the DFNRS and DNNNS sites in the AcrA acceptor protein^13^. The relative levels of glycosylation detected under these assay conditions varied, with the highest levels corresponding to OSTs from *C. jejuni*, *C. lari*, *C. gracilis* (PglB1), *W. succinogenes*, *H. pullorum* NCTC12824 (PglB1), and *D. gigas*.

### A subset of PglB homologs recognizes a non-canonical AQNAT motif

To determine whether any of the candidate OSTs could recognize a non-canonical AQNAT acceptor site, we tested each for glycosylation of scFv13-R4^AQNAT^, in which alanine is substituted in the −2 position of the C-terminal acceptor motif^15^. Glycosylation of the AQNAT sequon was clearly observed for 5 of the OSTs, namely *C. coli* PglB (*Cc*PglB), *C. upsaliensis* PglB (*Cu*PglB), *D. vulgaris* (*Dv*PglB), *Dg*PglB, and *Dd*PglB, as evidenced by the appearance of two protein bands in anti-His immunoblots and the detection of monoglycosylated scFv13-R4^AQNAT^ by hR6 antiserum (Fig. 1c). A faint band was also apparent for *Cj*PglB but only on the hR6 immunoblot, indicating a low level of non-consensus glycosylation. Similar promiscuous specificity has been occasionally observed for this OST and is dependent on the target protein and expression conditions used^15^. As with DQNAT, the relative levels of AQNAT glycosylation varied with the different OSTs. Overall, the strongest immunoblot signals corresponded to *Dg*PglB, which appeared to recognize AQNAT and DQNAT sites equally well as judged by the nearly equivalent amounts of glycosylated and aglycosylated protein in the anti-His immunoblot. *Dd*PglB showed similar levels of AQNAT glycosylation compared to *Dg*PglB, but appeared to prefer AQNAT over DQNAT.

It is noteworthy that all three of the *Desulfovibrio* OSTs produced an additional slower migrating band on the hR6 immunoblots, which corresponded to a diglycosylated form of scFv13-R4^(D/A)QNAT^ (Fig. 1b,c). We hypothesized that this second band resulted from glycosylation of a non-canonical N-X-S/T motif located within the scFv13-R4 protein. Two putative sites were identified: one at _32_FSNYS_36_ and another at _75_RDNAT_79_. When N77 was substituted with Leu in scFv13-R4^AQNAT^, only monoglycosylation was detected (SI Fig. S1). However, di-glycosylation was still detected with just an N34L substitution (SI Fig. S1). Therefore, in addition to the C-terminal AQNAT motif, the *Desulfovibrio* PglBs also recognized the internal non-canonical RDNAT motif in scFv13-R4.

### PglB homologs exhibit relaxed but different acceptor-site specificities

**Figure 2:**
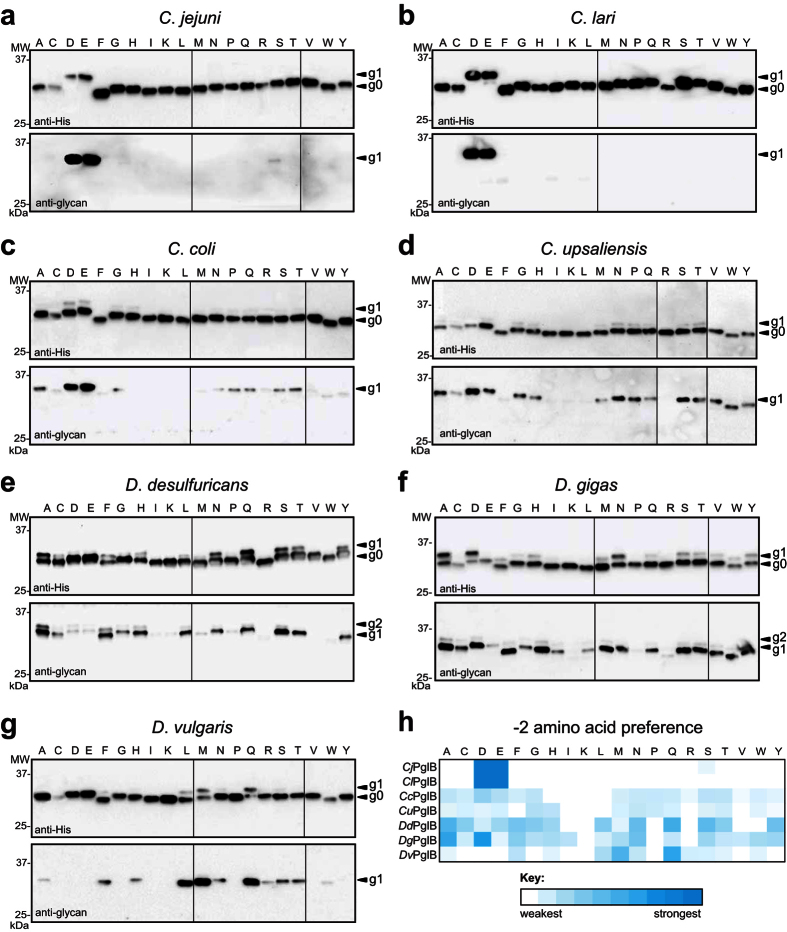
PglB homologs exhibit relaxed but different specificities for the −2 sequon residue. (**a**–**g**) Immunoblot analysis of TCA-precipitated periplasmic fractions from CLM24 cells complemented with one of the PglB homologs indicated, and co-expressing the biosynthetic pathway for the *C*. *jejuni* heptasaccharide and a panel of scFv13-R4^XQNAT^ variants (where X = one of the 20 amino acids). Blots were probed with anti-His antibodies against the C-terminal polyhistidine tag on acceptor protein or anti-glycan hR6 serum reactive with the *C*. *jejuni* heptasaccharide. The −2 residue in each acceptor protein is indicated across the top of each lane. The g0, g1, and g2 arrows indicate un-, mono-, and diglycosylated acceptor proteins, respectively. Molecular weight (MW) markers are indicated on the left. Due to the number of samples analyzed, all immunoblots shown are composites, delineated by dividing lines, from similar exposures. (**h**) Heatmap analysis of the relative −2 amino acid preference of each OST in (**a**–**g**). Relative preferences (weaker = white; stronger = dark cyan) were determined based on densitometric quantification of the percent glycosylated (defined as g1/g0 ratio) for each acceptor protein in the anti-His immunoblot.

Next, we systematically probed the −2 position acceptor sequon specificity of the 5 OSTs that catalyzed non-canonical AQNAT glycosylation. This involved a previously constructed set of plasmids encoding scFv13-R4 acceptor proteins in which the −2 position of the C-terminal acceptor motif was varied to include all 20 amino acids^15^. Control cells complemented with *Cj*PglB or *Cl*PglB showed the expected preference for D/E in the −2 position when tested against the panel of scFv13-R4^XQNAT^ acceptor-site variants (Fig. 2a,b). In contrast to the narrow specificity of *Cj*PglB and *Cl*PglB, the *Cc*PglB, *Cu*PglB, *Dd*PglB, *Dg*PglB, and *Dv*PglB enzymes all tolerated a wide range of amino acid substitutions in the −2 position of the acceptor site, albeit to varying extents (Fig. 2c–f). The −2 position amino acid preferences observed for these OSTs are summarized by heatmap analysis (Fig. 2h). *Cc*PglB exhibited preferences for the canonical DQNAT and EQNAT motifs, glycosylating these targets to relatively higher levels than any of the non-canonical sequons. *Cu*PglB showed no clear bias for any particular sequon, glycosylating nearly all acceptor sites (15 out of 20) with comparable efficiency. *Dd*PglB also glycosylated the majority of acceptor sites (15 of the 20) with the highest levels observed for AQNAT, QQNAT, and SQNAT motifs. *Dv*PglB exhibited a unique preference profile for LQNAT, MQNAT and QQNAT and barely detectable glycosylation of the canonical D/EQNAT sequons. *Dg*PglB exhibited the broadest specificity of all the OSTs tested, recognizing every sequon except for KQNAT, PQNAT and RQNAT. In fact, *Dg*PglB glycosylated two non-canonical sequons, AQNAT and NQNAT, as efficiently as the canonical DQNAT sequon. As above, *Dd*PglB and *Dg*PglB produced diglycosylated scFv13-R4^XQNAT^ proteins that were attributed to internal glycosylation of residue N77. Glycosylation of N77, an RDNAT sequon, but lack of RQNAT glycosylation indicated these OSTs can accommodate Arg in the −2 position but with an apparent stronger dependence on its context.

### Structural modeling of *Desulfovibrio* sp. PglB homologs

**Figure 3:**
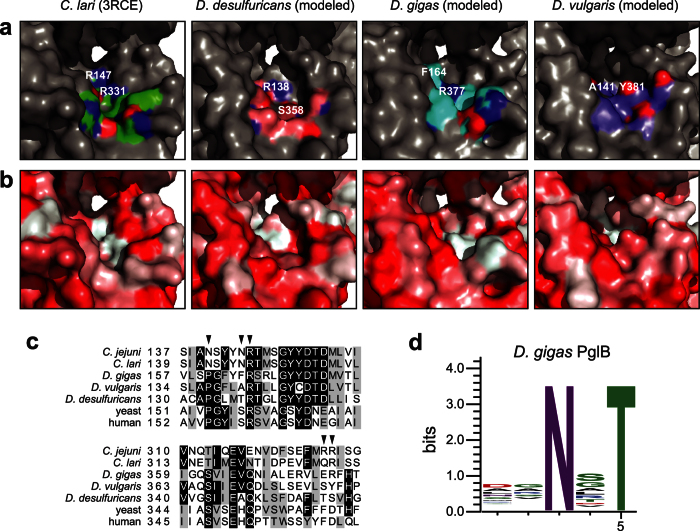
Molecular determinants of relaxed acceptor-site specificity. (**a**,**b**) Homology models of *Dd*PglB, *Dg*PglB, and *Dv*PglB compared to the crystal structure of *Cl*PglB (pdb code: 3RCE). Differences in the presence and distribution of polar and electrostatic (**a**) and hydrophobic surfaces (**b**) are colored. Backbone residues are colored in green (*C*. *lari*), pink (*D*. *desulfuricans*), cyan (*D*. *gigas*), and purple (*D*. *vulgaris*). Red and blue shading indicates oxygen and nitrogen atoms, respectively. Conserved *Cl*PglB residue R331 and corresponding residues in *Dd*PglB (S358), *Dg*PglB (R377) and *Dv*PglB (Y381) are labeled. Conserved *Cl*PglB residue R147 and its corresponding residue in *Dd*PglB (R138) are labeled, but the homologous residues in *Dg*PglB (R165) and *Dv*PglB (R142) are not visible in the pocket. Adjacent residues F164 and A141 in *Dg*PglB and *Dv*PglB, respectively, are labeled. (**c**) Multiple sequence alignment of 5 PglB homologs compared to 2 eukaryotic STT3 sequences using Clustal Omega. Sequences span regions homologous to *Cl*PglB residues 139–160 and 313–334. Shading indicates residue conservation. Some residues of interest are indicated with arrows across the top. (**d**) Sequence logo showing experimentally determined acceptor-site specificity of *Dg*PglB using glycoSNAP-based library screening of YebF-R4Δ1-198^XXNXT^.

To better understand the relaxation of the *Desulfovibrio* sp. OSTs from a structural perspective, homology models of each were derived using I-TASSER and compared to the X-ray crystal structure of *Cl*PglB^6^, an OST having narrow substrate specificity (see Fig. 2b). The overall backbone structures of each modeled enzyme were visually similar to *Cl*PglB, and sequence-independent structural alignment confirmed this, showing root-mean-square deviations (RMSD) of 0.440, 1.165, and 1.006 for *Dd*PglB, *Dg*PglB, and *Dv*PglB, respectively, where high structural similarity is considered an RMSD <2^24^. However, there were distinct differences in the predicted surfaces of the peptide/protein-binding cavities relative to *Cl*PglB, and each other. Changes in both the cavity accessibility and in the residues lining each cavity could alter the polar, electrostatic, and hydrophobic interactions that might stabilize the acceptor substrate (Fig. 3a,b). In *Cl*PglB, a salt bridge between residue R331 and the −2 Asp of a bound acceptor peptide is proposed to strengthen the PglB-peptide interaction and serves as a key determinant of the more specific site selection by bacterial OSTs^6^. In line with this observation, there is no occurrence of Arg in the modeled peptide/protein-binding cavity of *Dv*PglB (Fig. 3a), which exhibited no apparent glycosylation of (D/E)QNAT. *Dd*PglB, which weakly glycosylated (D/E)QNAT but preferred non-acidic residues in the −2 position, was predicted to have the most open/accessible cavity and only retained R138 in this cavity (Fig. 3a), which corresponds to conserved *Cl*PglB R147 (Fig. 3c). Interestingly, the peptide/protein-binding cavity of the most relaxed homolog, *Dg*PglB, still involved R377 (Fig. 3a), which is equivalent to *Cl*PglB R331 (Fig. 3c), suggesting that additional determinants more strongly influence acceptor peptide selection by this enzyme.

### Structural analysis of *in vivo*-generated glycoproteins

To confirm glycosylation of non-canonical acceptor sites by the different PglB homologs, we performed mass spectrometry using scFv13-R4^DQNAT^ and scFv13-R4^AQNAT^ as acceptor proteins. A trypsin site (Gly-Lys-Gly) immediately after the glycosylation tag facilitates removal of the positively charged 6x-His tag^15^. The scFv13-R4^DQNAT^ and scFv13-R4^AQNAT^ constructs were glycosylated in cells complemented with one of the PglB homologs, after which glycoproteins were purified using nickel-affinity chromatography, treated with trypsin, and subjected to liquid chromatography-mass spectrometry (LC-MS) analysis. Consistent with the *in vivo* experiments above, *Cc*PglB, *Cu*PglB, and *Dv*PglB all transferred the 1405.56-Da *C. jejuni* heptasaccharide with bacillosamine as the innermost saccharide to the N273 residue in either scFv13-R4^DQNAT^ or scFv13-R4^AQNAT^ (SI Figs. S2 and S3). Since *Dv*PglB-mediated glycosylation of scFv13-R4^AQNAT^ was undetectable in Coomassie-stained gels, we instead used a more efficiently glycosylated acceptor protein variant, scFv13-R4^QQNAT^ (SI Fig. S4a). LC-MS of gel-extracted tryptic digests of the purified protein demonstrated that *Dv*PglB transferred the *C. jejuni* glycan to N273 in scFv13-R4^QQNAT^ (SI Fig. S4b). Interestingly, a glycopeptide bearing a mass of 1380.56 Da was also identified on N273 in scFv13-R4^QQNAT^ (SI Fig. S4c). This different glycoform was later determined by the MS/MS spectra to contain a HexNAc in place of Bac as the innermost monosaccharide unit of the glycan structure. Such an occurrence has previously been reported for *Cj*PglB^16^. Collectively, these results unambiguously confirm glycan attachment to the non-consensus AQNAT (or QQNAT) site by all of the PglB homologs tested.

### Relaxed PglB homologs glycosylate non-canonical sites *in vitro*

Next, we adapted a recently developed *in vitro* glycosylation assay^25^ to further characterize the PglB homologs having relaxed specificity. Specifically, purified scFv13-R4 (N34L,N77L)^DQNAT^ or scFv13-R4 (N34L,N77L)^AQNAT^ was incubated with detergent-solubilized membrane preparations from *E. coli* cells expressing one of the PglB homologs along with lipid-linked GalNAc_5_(Glc)Bac glycans extracted separately from *E. coli* cells expressing the entire *C. jejuni pgl* pathway except for *Cj*PglB. Under the conditions tested here, all of the OSTs except *Dv*PglB showed detectable *in vitro* glycosylation of the canonical sequon in scFv13-R4 (N34L,N77L)^DQNAT^ whereas only *Dd*PglB and *Dg*PglB, and to a much lesser extent *Cj*PglB, glycosylated the non-canonical sequon in scFv13-R4 (N34L,N77L)^AQNAT^ (SI Fig. S5a). While most of the *in vitro* results corroborated our *in vivo* observations, the lack of scFv13-R4 (N34L,N77L)^AQNAT^ glycosylation by *Cc*PglB, *Cu*PglB and *Dv*PglB was unexpected. To rule out any issues with the use of a folded acceptor protein, we decided to also test a minimal peptide substrate. This involved adapting an *in vitro* assay developed for analysis of OST activity^26^. Here, we incubated PglB-containing membranes and lipid-linked GalNAc_5_(Glc)Bac glycans with an 8-mer peptide (either GDQNATAF or GAQNATAF) labeled at the N-terminus with the fluorescent dye TAMRA. After separation of the reaction products by Tris-Tricine/SDS-PAGE and detection of fluorescence signals, we observed that all OSTs except *Dd*PglB modified the TAMRA-GDQNATAF peptide containing the canonical sequon as evidenced by a shift in electrophoretic mobility (SI Fig. S5b). In contrast, *Dg*PglB was the only OST examined that glycosylated the TAMRA-GAQNATAF peptide bearing the non-canonical sequon (SI Fig. S5b).

To prove glycosylation of the non-canonical acceptor peptide directly, we analyzed the *in vitro* glycosylation product by LC-MS. The MS/MS spectrum on the triply-charged precursor (*m*/*z* 866.43) identified the glycopeptide and a heptasaccharide glycan (1405.56 Da) with bacillosamine as the innermost saccharide attached to the N4 residue in TAMRA-GAQNATAF (SI Fig. S5c). These results unequivocally confirm non-consensus glycosylation by *Dg*PglB.

### Unbiased definition of *Dg*PglB sequon specificity

In light of its relaxed acceptor-site specificity both *in vivo* and *in vitro*, we decided to analyze acceptor-site specificity of the *Dg*PglB enzyme in a more unbiased manner. This involved using our previously established genetic assay named glyco-SNAP (glycosylation of secreted *N*-linked acceptor proteins), a high-throughput colony blotting assay based on glycosylation and extracellular secretion of YebF modified with an acceptor sequon^15^. To obtain signal above background in this assay, secreted glycoprotein levels must be readily detected on an anti-His immunoblot. Since the level of *Dg*PglB-mediated glycosylation of YebF^AQNAT^ was too low for this assay (SI Fig. S6a), we adapted the glycoSNAP assay by creating chimeras between YebF and either the full-length scFv13-R4^AQNAT^ acceptor protein or a truncated acceptor comprised of the last 72 amino acids of scFv13-R4 (scFv13-R4Δ1-198^AQNAT^). Both were tested for glycosylation and secretion in the presence of *Dg*PglB. While the overall accumulation of scFv13-R4Δ1-198^AQNAT^ in culture supernatants was lower, the glycosylation efficiency as assessed by anti-His immunoblot was sufficient for glycoSNAP screening (SI Fig. S6b); hence, we opted to proceed with this chimeric acceptor protein. A 7.7 × 10^5^-member combinatorial library of scFv13-R4Δ1-198^XXNXT^ variants was generated by PCR using degenerate primers with NNK bases at the codons for the −2, −1, and +1 target sequon positions. The resulting YebF(N24L)-R4Δ1-198^XXNXT^ library was screened for sequons glycosylated by *Dg*PglB. A total of 31 positive hits were recovered and used to generate a sequence logo of *Dg*PglB acceptor-site preferences (Fig. 3d). Overall, *Dg*PglB exhibited relaxed specificity at all three variable sequon positions, with a slight bias at the +1 sequon position, where Ser was picked 7 times. Asp and Ala were picked most for the −2 position, 5 and 4 times respectively. This result agreed well with the results above, where the most efficient glycosylation by *Dg*PglB occurred with the scFv13-R4^DQNAT^ and scFv13-R4^AQNAT^ proteins (see Fig. 2f).

### Glycosylation of native human IgG1 Fc domain by *Dg*PglB

**Figure 4:**
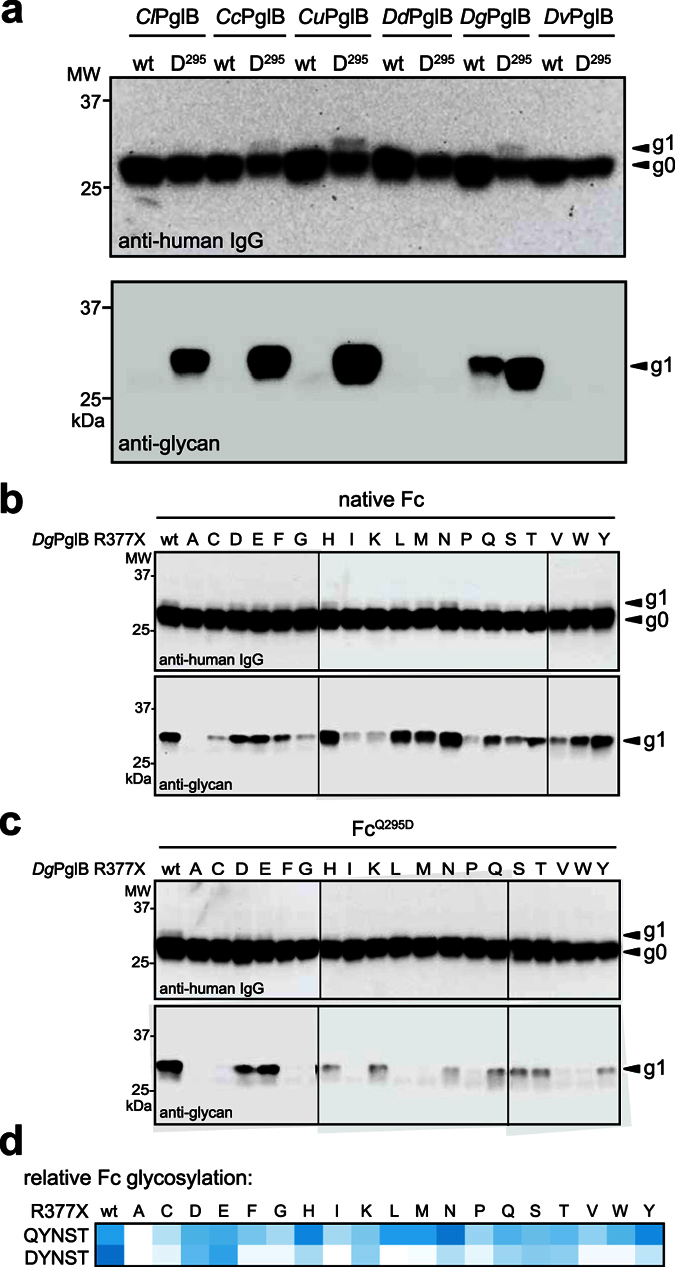
Glycosylation of the native human IgG Fc domain by PglB homologs. (**a**) Immunoblot analysis of TCA-precipitated periplasmic fractions from CLM24 cells complemented with one of the PglB homologs as indicated, and co-expressing the biosynthetic pathway for the *C*. *jejuni* heptasaccharide and either native Fc domain (wt) or the Fc^Q295D^ mutant (D^295^). (**b**,**c**) Same as in (**a**) but cells were complemented with a site-directed *Dg*PglB R377X mutant (where X = one of the 20 amino acids). Blots in (**a**–**c**) were probed with anti-human IgG or anti-glycan hR6 serum reactive with the *C*. *jejuni* heptasaccharide. The g0 and g1 arrows indicate un- and monoglycosylated acceptor proteins, respectively. Immunoblots shown in (**b**,**c**) are composites, delineated by the dividing lines, from similar exposures based on the same wt sample loaded on all gels (not shown). Molecular weight (MW) markers are indicated on the left. (**d**) Heatmap analysis of relative Fc glycosylation levels by each site-directed *Dg*PglB mutant. Relative glycosylation site preferences (weaker = white; stronger = dark cyan) was determined based on densitometry of the anti-glycan immunoblot bands, normalized to the sample corresponding to Fc glycosylation by wt *Dg*PglB run on the same gels and included in each separate experiment.

Given their relaxed specificity, we hypothesized that one or more of the relaxed PglB homologs might recognize a short eukaryotic N-X-S/T glycosylation site in a native human glycoprotein. To test this notion, these select OSTs were evaluated for the ability to glycosylate the Fc domain of human immunoglobulin G (IgG), which contains a single conserved glycosylation site at N297 in the sequon QYNST. Consistent with earlier findings^8^,^17^,^27^,^28^, *Cl*PglB glycosylated an Fc^Q295D^ mutant that was modified to contain an acidic residue in the −2 position of the sequon; however, this OST was unable to glycosylate the wild-type (wt) Fc domain (Fig. 4a), consistent with earlier findings^17^. Even a panel of relaxed *Cj*PglB variants, in which DL, NL, or LQ were substituted for R327 and R328^15^, failed to detectably glycosylate the native QYNST site of the wt Fc domain (SI Fig. S7a). A similar result was observed for the more relaxed OSTs from *Campylobacter* sp., *Cc*PglB and *Cu*PglB, albeit with the latter OST furnishing the highest level of Fc^Q295D^ glycosylation. The OSTs from *Desulfovibrio* sp. showed profiles that were distinct from the *Campylobacter* OSTs and from each other. *Dd*PglB did not detectably glycosylate either wt Fc or Fc^Q295D^ (Fig. 4a), although very weak glycosylation of wt Fc became apparent after long exposures. *Dv*PglB was unable to glycosylate either of the Fc domains (Fig. 4a). *Dg*PglB, on the other hand, showed the unique ability to glycosylate both the Fc^Q295D^ and the wt Fc domain (Fig. 4a). This ability was not solely attributed to the residues corresponding to *Cj*PglB R327 and R328 (E376 and R377 in *Dg*PglB) as *Dg*PglB NL and LQ mutants retained the ability to glycosylate the wt Fc domain (SI Fig. S7b). This work provides the first example of native Fc glycosylation in a bacterial system.

### Rationally designed *Dg*PglB variants exhibit increased Fc glycosylation

As discussed above, residue R331 in *Cl*PglB has been proposed to play a direct role in site selection by bacterial OSTs^6^,^29^. Indeed, mutations to the corresponding residue in *Cj*PglB, R328, together with the neighboring upstream residue yielded OST variants with greatly relaxed acceptor-site specificity compared to the parental enzyme^15^. In light of these findings, we hypothesized that while the presence of the conserved R377 residue in *Dg*PglB (equivalent to *Cl*PglB R331; see Fig. 3c) did not restrict acceptor site selection, it was still a key determinant in modulating glycosylation levels of a given acceptor sequon. The relatively low level of Fc glycosylation detected also revealed *Dg*PglB as a prime candidate for rational engineering to improve Fc glycosylation efficiency. As proof-of-concept, we performed site-saturation mutagenesis of *Dg*PglB to generate a library of *Dg*PglB variants having all possible amino acids substituted at position R377. The resulting library was screened for variant OSTs having the ability to glycosylate the QYNST and DYNST sequons in the native Fc and Fc^Q295D^, respectively. Unlike the parental *Dg*PglB enzyme that preferred DYNST over QYNST, *Dg*PglB variants with His, Asn, and Tyr substituted for Arg resulted in both increased preference for the native QYNST site over DYNST, and also slightly increased relative glycosylation efficiency of the QYNST site (Fig. 4b,c). Almost all mutations resulted in a decrease in Fc^Q295D^ glycosylation, as summarized by heatmap analysis (Fig. 4d). Interestingly, reversing the charge by replacement of Arg with Asp or Glu resulted in nearly identical apparent levels of glycosylation for the native Fc and Fc^Q295D^ (Fig. 4b,c) and the highest levels of Fc^Q295D^ glycosylation among the 19 mutant enzymes (Fig. 4c). This result suggested that salt bridge formation between residue R377 and the −2 residue of the sequon does not contribute to *Dg*PglB-peptide interaction as it does in the case of *Cl*PglB^6^, as the like charge would otherwise be expected to be unfavorable to acceptor peptide binding or recognition. Overall, the influence of *Dg*PglB R377 appears to be dominated by polar interactions, with hydrophobic interactions also having noticeable influence with the native Fc acceptor sequon.

## Discussion

The acceptor-site specificity of the prototypical bacterial OST, *Cj*PglB, is generally restricted to motifs having an acidic residue in the −2 position relative to the target Asn^10^. To date, only minor exceptions to this so-called minus two rule for *Cj*PglB have been reported whereby glycosylation was observed at a single non-canonical site in a specific bacterial target protein^13^,^17^. Here, we show that 5 bacterial homologs having both high (>65%) and low (<25%) sequence identity to *Cj*PglB have much different acceptor-site preferences compared to *Cj*PglB and to each other, with each glycosylating targets having diverse amino acids in the −2 position of the target sequon. One of these single-subunit bacterial OSTs, namely *Dg*PglB, exhibited functional specificities that overlapped with eukaryotic STT3.

At present, the rules defining substrate specificity in these naturally relaxed OSTs, as well as in previously engineered *Cj*PglB variants with relaxed specificity^15^, remain poorly defined. Multiple sequence alignments showed that *Cc*PglB, *Cu*PglB, and *Dg*PglB all retain the bacterially conserved Arg, equivalent to *Cl*PglB R331, indicating that while this residue appears to be involved in acceptor protein recognition or binding^6^,^15^,^29^, it is not necessarily the primary factor governing the minus two rule. It is possible that the peptide/protein binding cavities of these relaxed OSTs are remodeled in such a way that this Arg residue is repositioned in the context of acceptor-site binding. Its role, however, is not completely eliminated, as substitutions at this site in *Dg*PglB still conferred changes in Fc glycosylation levels. These results may also support a possible role for the conserved Arg corresponding to *Cl*PglB R147^6^,^29^ and suggest the potential importance of nearby residues. All three *Desulfovibrio* sp. PglBs have a Pro substituted five residues upstream of the homologous R147 residue, which could potentially reposition residues into or out of the binding cavity. Pro is notably conserved in this position in eukaryotic STT3s. In our model, *Dg*PglB also has a bulky aromatic residue, F164, immediately adjacent to the R147 homologous residue, effectively replacing its surface space in the protein-binding cavity.

While acceptor-site specificity of bacterial OSTs appears to involve two key Arg residues in the peptide/protein-binding cavity, clearly there are still other specificity determinants present in these enzymes. It is also important to note that there may not be a “one size fits all” bacterial OST, as relaxation toward the −2 position in the sequon did not necessarily correlate with broad abilities to glycosylate native eukaryotic glycoproteins. Indeed, only *Dg*PglB and to a low level *Dd*PglB were able to detectably glycosylate the native glycosylation site in the Fc domain of human IgG. Some of the relaxed variants were able, however, to glycosylate the Fc domain when an acidic residue was substituted in the minus two position, suggesting these OSTs were able to recognize the glycosylation site but perhaps require additional stabilization afforded by the introduced electrostatic or polar interactions for successful catalysis. The fact that relaxed bacterial OSTs exhibit different profiles for native glycosylation site preferences may be advantageous for the glycosylation of biotherapeutics, where the natural preference introduces a novel level of control over glycan site occupancy.

Overall, these results present, to date, the most comprehensive analysis of single-subunit bacterial OSTs and provide novel insights into bacterial *N*-linked glycosylation, showing that the generally accepted “minus two rule” for acceptor-site specificity does not hold true for all bacterial *N-*glycosylation catalysts. Nonetheless, distinct site preferences are still present and remain to be identified. The discovery of the first bacterial OST capable of glycosylating the native Fc domain in human IgG opens the door to a bacterial pathway for antibody glycoengineering. While further work is needed to increase efficiency, we show that glycosylation levels of the Fc domain can be improved through rational design of improved *Dg*PglB biocatalysts.

## Materials and Methods

### Bacterial strains and growth conditions

*E. coli* strain DH5α was used for all cloning and site-directed mutagenesis. *E. coli* strain CLM24^7^ was used for all *in vivo* glycosylation studies. *E. coli* strain BL21(DE3) was used to produce acceptor proteins for *in vitro* glycosylation studies. *E. coli* strain SCM6^30^ was used for preparation of LLOs while either C43(DE3)^31^ or CLM24 cells were used to prepare solubilized membrane extracts containing OSTs, as described below. Cultures were grown at 37 °C in Luria-Bertani (LB) broth supplemented with 0.2% (w/v) D-glucose and antibiotics as needed at the following concentrations: 100 μg/ml ampicillin (Amp), 20 μg/ml chloramphenicol (Cm), and 80 μg/ml spectinomycin (Spec). Protein expression was induced by 0.2% (w/v) L-arabinose for pBAD-based expression vectors and 0.1 mM isopropyl β-D-thiogalactoside (IPTG) for pTrc99-based expression vectors. Cultures were typically induced at mid-log phase for 16–20 h at 30 °C.

### Plasmid construction

Plasmid pACYC*pglB::kan* is a pACYC184-based plasmid encoding the *C.jejuni pgl* locus with the *pglB* gene replaced by a kanamycin resistance cassette^23^. The pSF-OST plasmids were constructed by separately cloning each of the 24 bacterial OST genes into plasmid pSF^15^ between the XbaI and SbfI (e.g., plasmid pSF-*Cj*PglB for *C. jejuni* OST; pSF-*Dg*PglB for *D. gigas* OST). Template DNA for bacterial OSTs was obtained from ATCC or provided as a generous gift from Dr. Bil Clemons (California Institute of Technology). Plasmids encoding the R377X variants were generated by site-directed mutagenesis of plasmid pSF-*Dg*PglB using either NNK degenerate primers followed by sequencing to identify individual substitutions or primers encoding the base changes for the desired substitution. Plasmids pBS-scFv13-R4^XQNAT^ and pBS-scFv13-R4^AQNAT-GKG-His6^ were described previously^15^. To facilitate mass spectrometry analysis, plasmids pBS-scFv13-R4^(D/Q)QNAT-GKG-His6^ were generated by inserting the codon for Lys between the existing two codons for the Gly linker by site-directed mutagenesis of the parental pBS-scFv13-R4^(D/Q)QNAT^ plasmids. Plasmids pBS-scFv13-R4(N34L)^AQNAT^, pBS-R4(N77L)^AQNAT^, and pBS-R4(N34L, N77L)^AQNAT^ were constructed by site directed mutagenesis, replacing the codons for Asn with a codon for Leu to eliminate putative internal sequons. Plasmid pET28a-scFv13-R4(N34L, N77L)^(D/A)QNAT-His6^ was constructed by subcloning DNA encoding scFv13-R4(N34L, N77L)^(D/A)QNAT-His6^ from pBS-R4(N34L,N77L)^(D/A)QNAT-His6^ between the NcoI and HindIII sites in pET28a(+). pTrc99A-YebF(N24L)-scFv13-R4(N34L, N77L)^AQNAT^ was constructed by cloning the DNA encoding scFv13-R4(N34L, N77L)^AQNAT-His6^ between the XbaI and HindIII sites of pTrc99A-YebF(N24L)1xAQNAT-3xNAV 15, replacing the glycosylation tag and polyhistidine epitope tag in this parental plasmid. For compatibility with the other plasmids used in this study, DNA encoding YebF(N24L)-R4(N34L, N77L)^AQNAT-His6^ was subsequently subcloned into the NcoI and HindIII sites of the pTrc99S plasmid, described below. Plasmid pTrc99S-YebF(N24L)-scFv13-R4Δ1-198^AQNAT^ was made by in-frame deletion of codons for all but the last 72 amino acids of scFv13-R4 using extra-long PCR with 5′ phosphorylated primers and ligation of the resulting linear PCR products. The 7.7 × 10^5^-member combinatorial library of pTrc99S- YebF(N24L)-scFv13-R4Δ1-198^XXNXT^ was constructed by site-directed mutagenesis using degenerate primers with NNK bases (N = A, C, T or G; K = G or T) at the −2, −1, and +1 positions of the glycosylation tag sequon. Plasmid pTrc99S (Spec^r^) was generated from pTrc99A (Amersham Pharmacia) by replacing the Amp resistance cassette with a Spec resistance cassette through homology assembly (Gibson Assembly Master Mix, New England Biolabs). DNA encoding fusions between the *E. coli* DsbA signal peptide and human IgG1 Fc domain (wt Fc) or Fc^Q295D ^27 was cloned into the pTrc99S plasmid between the EcoRI and HindIII sites. All plasmids were confirmed by DNA sequencing at the Cornell Biotechnology Resource Center.

### Protein analysis

To analyze products of *in vivo* glycosylation, periplasmic fractions were harvested after spheroplasting an equivalent amount of cells in buffer containing 0.2 M Tris-Ac (pH 8.2), 0.25 M sucrose, 160 μg/ml lysozyme, and 0.25 mM EDTA. Fractions were precipitated by addition of an equal volume of ice-cold 20% trichloroacetic acid. Resulting protein pellets were solubilized in Laemmli sample buffer containing 5% β-mercaptoethanol (βME) and resolved on SDS-polyacrylamide gels (BioRad). Western blotting used 6x-His tag-specific polyclonal antibodies (Abcam) or *C*. *jejuni* heptasaccharide glycan-specific antiserum hR6^17^. Pierce enhanced chemiluminescent (ECL) substrate (Thermo Scientific) was used for detection of bound antibodies. All blots were visualized using a Chemidoc^TM^ XRS+ system with Image Lab^TM^ image capture software (BioRad).

### *In vitro* glycosylation

Methods for purification of the scFv13-R4(N34L, N77L)^D/AQNAT^ acceptor protein and isolation of *C. jejuni* lipid-linked oligosaccharides (LLOs) were described previously^25^. Briefly, acceptor proteins were purified from BL21(DE3) cells carrying pET28a-scFv13-R4(N34L, N77L)^D/AQNAT-His6^ plasmid using standard Ni^2+^-affinity chromatography (HisTrap HP, GE Healthcare) followed by size exclusion chromatography with Superdex-75 columns (GE Healthcare). LLOs were extracted from SCM6 cells carrying plasmid pMW07-pglΔB^15^, which encodes the entire *C. jejuni pgl* pathway except for *Cj*PglB, by chloroform/methanol/water extraction. Preparation of solubilized membrane extracts containing candidate OSTs was performed as follows. C43 cells carrying a pSF-OST plasmid (encoding one of the candidate OSTs) were grown at 37 °C in LB media with shaking to mid-logarithmic phase, then induced with 0.2% arabinose and grown overnight at 30 °C. Cells were harvested by centrifugation at 10,000 × g for 15 min at 4 °C, resuspended in buffer 1 containing 50 mM Tris-HCl (pH 7.0) and 25 mM NaCl at 10 ml per gram of cell pellet, and frozen at −80 °C. For final processing, cells were gradually thawed on ice and then disrupted using an EmulsiFlex-C5 homogenizer (Avestin). Unbroken cells were removed by two rounds of centrifugation at 15,000 × g for 20 min at 4 °C. The supernatant was ultracentrifuged at 100,000 × g for 1 h at 4 °C. The resulting membrane pellet was resuspended in buffer 2 containing 50 mM Tris-HCl (pH 7.0), 25 mM NaCl, and 1% Triton X-100 (Sigma) at 2 ml per gram of starting cell pellet. Following a 1-h incubation at room temperature, detergent-solubilized samples were centrifuged at 16,000 × g for 1 h at 4 °C, and the supernatants were directly employed as crude membrane extracts. Cell-free glycosylation of purified scFv13-R4(N34L, N77L)^D/AQNAT^ was carried out in a 50-μL reaction volume containing 2 μg of purified acceptor protein, 10 μL of extracted LLOs, and 30 μL of OST-containing crude membrane extract in 10 mM HEPES (pH 7.5), 10 mM MnCl_2_, and 0.1% (w/v) n-Dodecyl-β-D-maltoside (DDM). The reaction solution was incubated at 30° C for 12 h. Reactions were stopped by adding Laemmli sample buffer containing 5% βME followed by boiling at 100° C for 15 min. Reaction products were analyzed by immunoblot following SDS-PAGE.

*In vitro* glycosylation of TAMRA-labeled peptide substrates^26^ was carried out using crude OST extracts prepared as described above but using CLM24 cells carrying pSF-OST plasmids encoding candidate OSTs, described above. Reactions were carried out in either 59-μL reactions containing 8.5 μM *N*-terminal TAMRA-labeled GDQNATAF peptide substrate (Thermo Fisher Scientific), supplemented with 5 mM MnCl_2_, or 66-μL reactions containing 18 μM *N*-terminal TAMRA-labeled GAQNATAF peptide substrate (Thermo Fisher Scientific), supplemented with 4.5 mM MnCl_2_. All reactions contained 2 μL of extracted LLOs and 50 μL of OST-containing crude membrane extracts, and were brought to volume with buffer containing 50 mM Tris-HCl (pH 7.0), 25 mM NaCl, and 1% Triton X-100. The reactions were incubated overnight at 30 °C with shaking. Reactions were stopped by adding Laemmli sample buffer containing 5% βME followed by boiling at 100 °C for 15 min. Reaction products were analyzed through Tris-Tricine SDS-PAGE followed by detection of fluorescence signals^32^.

### MS analysis

The scFv13-R4^DQNAT^, scFv13-R4^AQNAT^, and scFv13-R4^QQNAT^ acceptor proteins were co-expressed in cells complemented with candidate OSTs. 500-mL cultures were used to generate periplasmic fractions, followed by purification with Ni-NTA spin columns (Qiagen). Approximately 1 μg of protein was resolved on SDS-polyacrylamide gels and stained with Biosafe Coomassie stain (BioRad). The detected glycoprotein bands (~36 kDa) were excised and subjected to in-gel digestion with trypsin followed by extraction of the tryptic peptide as previously described^15^. Subsequent precursor ion scanning MS analysis (nanoLC-ESI-MS/MS) was carried out with UltiMate3000 nanoLC (Thermo/Dionex) coupled with a hybrid triple quadrupole linear ion trap 4000 Q Trap mass spectrometer, which was equipped with a Micro Ion Spray Head II ion source (AB SCIEX). MS data acquisition was performed using Analyst 1.4.2 software (AB SCIEX) for PI scan triggered information-dependent acquisition (IDA) analysis^33^. All acquired MS and MS/MS spectra triggered by PI scan on *m*/*z* 204 were manually inspected and interpreted with Analyst 1.4.2 and BioAnalysis 1.4 software (Applied Biosystems) for identification of the glycopeptide sequence, the *N*-linked glycosylation sites and glycan compositions.

### GlycoSNAP assay

The YebF(N24L)-scFv13-R4Δ1-198^XXNXT^ combinatorial library was screened using the previously established glycoSNAP assay^15^. Briefly, library transformants were plated on 150 mm LB agar plates containing 80 μg/ml Spec, 20 μg/ml Cm, 100 μg/ml Amp, and 0.2% (w/v) D-glucose and incubated overnight at 37 °C. Colonies were replicated the second day on circles of Whatman 0.45-μm 142-mm cellulose nitrate membrane filters (VWR), overlayed on nitrocellulose transfer membrane (Fisher Scientific) on an induction plate consisting of LB agar containing 80 μg/ml Spec, 20 μg/ml Cm, 100 μg/ml Amp, 0.1 mM IPTG, and 0.2% (w/v) L-arabinose, and incubated at 30 °C for 16–20 h. The nitrocellulose membranes were then immunoblotted using *C*. *jejuni* heptasaccharide glycan-specific antiserum hR6. Signal was matched to corresponding colonies preserved on the filter, and potential positive library screening hits were individually picked and re-streaked on LB agar plates containing 80 μg/ml Spec, 20 μg/ml Cm, 100 μg/ml Amp, and 0.2% (w/v) D-glucose before further analysis. All positive hits were confirmed by standard liquid culture growth, induction, and immunoblotting of periplasmic fractions, as described for protein glycosylation analysis above.

### Generation of molecular models and sequence logos

The I-TASSER server^34^,^35^,^36^ was used to predict the structure of *Dd*PglB, *Dg*PglB, and *Dv*PglB through a multiple threading algorithm. Structure prediction was initiated with no structural constraints. Structure images were generated using PyMOL Molecular Graphics System, Version 1.7.0.1 Schrödinger, LLC. Sequence logos were made from sequons of 30 confirmed positive hits from glycoSNAP screening of the YebF(N24L)-scFv13-R4Δ1-198^XXNXT^ library and generated using WebLogo 3^37^. Sequence conservation at each position was indicated by the height of each stack. Within each stack, the height of each amino acid letter represented its relative frequency at that position.

## Additional Information

**How to cite this article**: Ollis, A. A. *et al.* Substitute sweeteners: diverse bacterial oligosaccharyltransferases with unique N-glycosylation site preferences. *Sci. Rep.* **5**, 15237; doi: 10.1038/srep15237 (2015).

## Supplementary Material

Supplementary Information
