# Supplementary material for: Substitute sweeteners: diverse bacterial oligosaccharyltransferases with unique N-glycosylation site preferences
Source: Sci Rep. 2015 Oct 20;5:15237. doi: 10.1038/srep15237 (PMC4894442; doi:10.1038/srep15237)
Supplement: Supplementary Information [file srep15237-s1.pdf]

Supplementary Information File

Substitute sweeteners: diverse bacterial oligosaccharyltransferases with unique *N*-glycosylation site preferences

Anne A. Ollis, Yi Chai, Aravind Natarajan, Emily Perregaux, Thapakorn Jaroentomeechai, Cassandra Guarino, Jessica Smith, Sheng Zhang and Matthew P. DeLisa

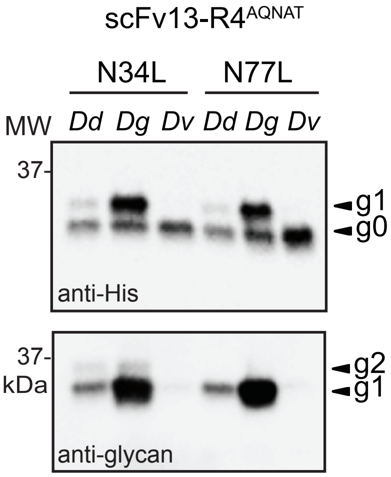

***SI* Figure S1. Glycosylation of residue N77 within non-canonical motif of scFv13-R4.** (a) Western blot analysis of scFv13-R4^{AQNAT} glycosylation by *Dd*PglB, *Dg*PglB or *Dv*PglB. Blots were probed with anti-His antibody (top panel) or hR6 antiserum containing *C. jejuni* heptasaccharide glycan-reactive polyclonal antibodies (bottom panel). The g0, g1, and g2 labels denote un-, mono-, and diglycosylated forms of scFv13-R4^{AQNAT}, respectively. The slower migrating band on anti-His immunoblot is the monoglycosylated form of scFv13-R4^{AQNAT}, confirmed by the anti-glycan immunoblot. Molecular weight (MW) markers are indicated at left. Results are representative of at least three biological replicates.

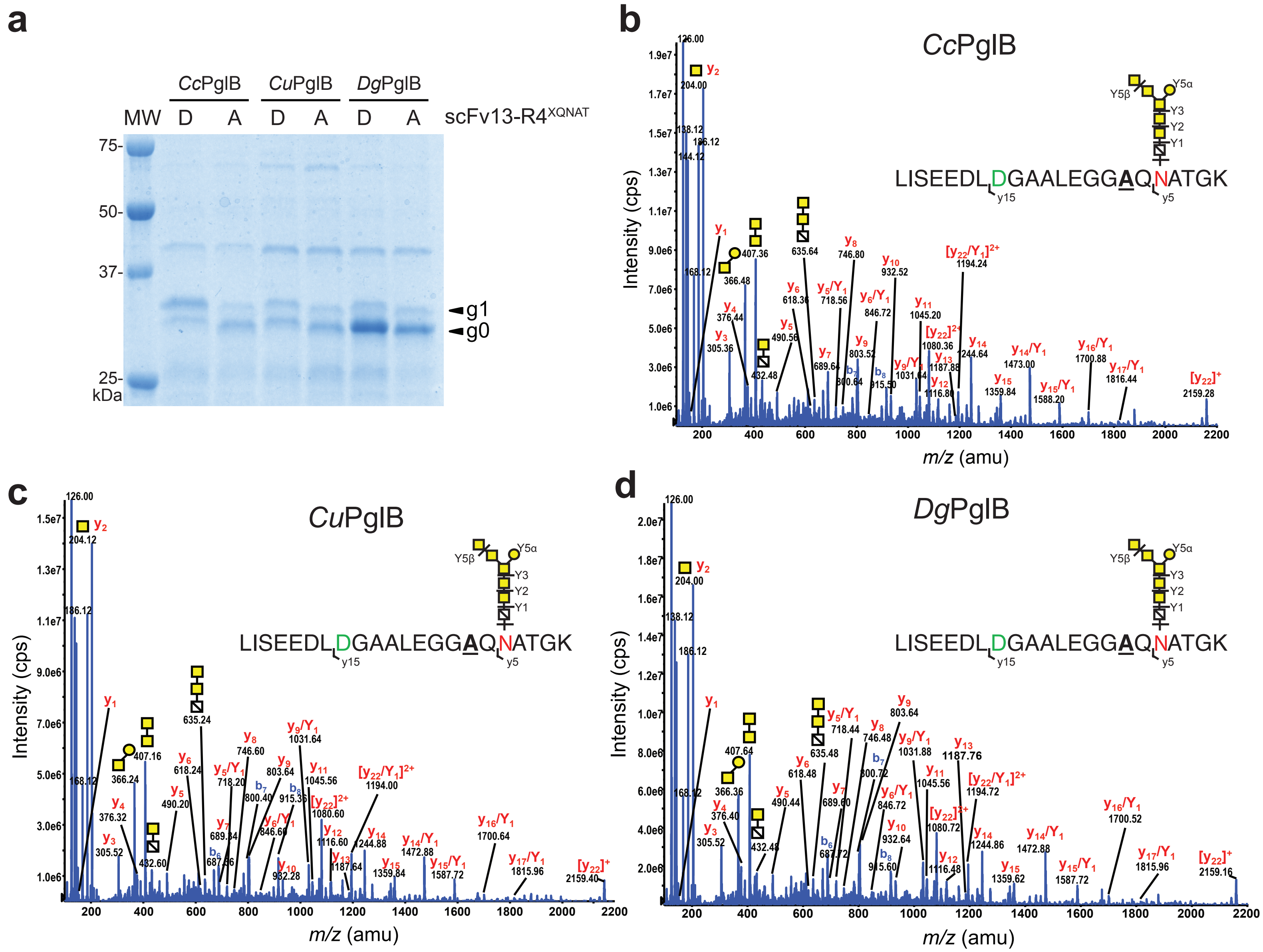

***SI* Figure S2. Mass spectrometry analysis of non-canonical glycosylation by *Cc*PglB, *Cu*PglB and *Dg*PglB.** (a) Ni-NTA-purified scFv13-R4^{DQNAT} and scFv13-R4^{AQNAT} samples used in MS analysis, stained with Coomassie Brilliant Blue G-250. Glycosylated bands, indicated by g1 arrow, were excised and submitted for MS analysis. Molecular weight (MW) markers are indicated at left. MS/MS spectrum of the triply-charged precursor ion for: (b) *Cc*PglB (*m/z* 1189.08); (c) *Cu*PglB (*m/z* 1188.97); and (d) *Dg*PglB (*m/z* 1189.05). These spectra identified the glycopeptide and a 1405.56 Da glycan with bacillosamine as the innermost saccharide attached to the N273 residue (shown in red) in scFv13-R4^{AQNAT}. In each case, a series of y-ions covering from y1 to y15 were observed with the complete knockout of glycan molecule, leading to the confident identification of tryptic peptide: 256-LISEEDLDGAALEGGAQNATGK-277, in which N263 was also found deamidated to Asp, consistent with common deamidation at an Asn residue followed by Gly residue. A second series of y-ions with the added mass of 228.11 Da at N273 site was also found covering from y9/Y1 to y17/Y1, providing direct evidence for bacillosamine as the innermost saccharide (Y1) attached to N273. These results are also consistent with the previous observation that a relatively tight bond exists for Y1-peptide compared to the fragile internal glycan bonds.

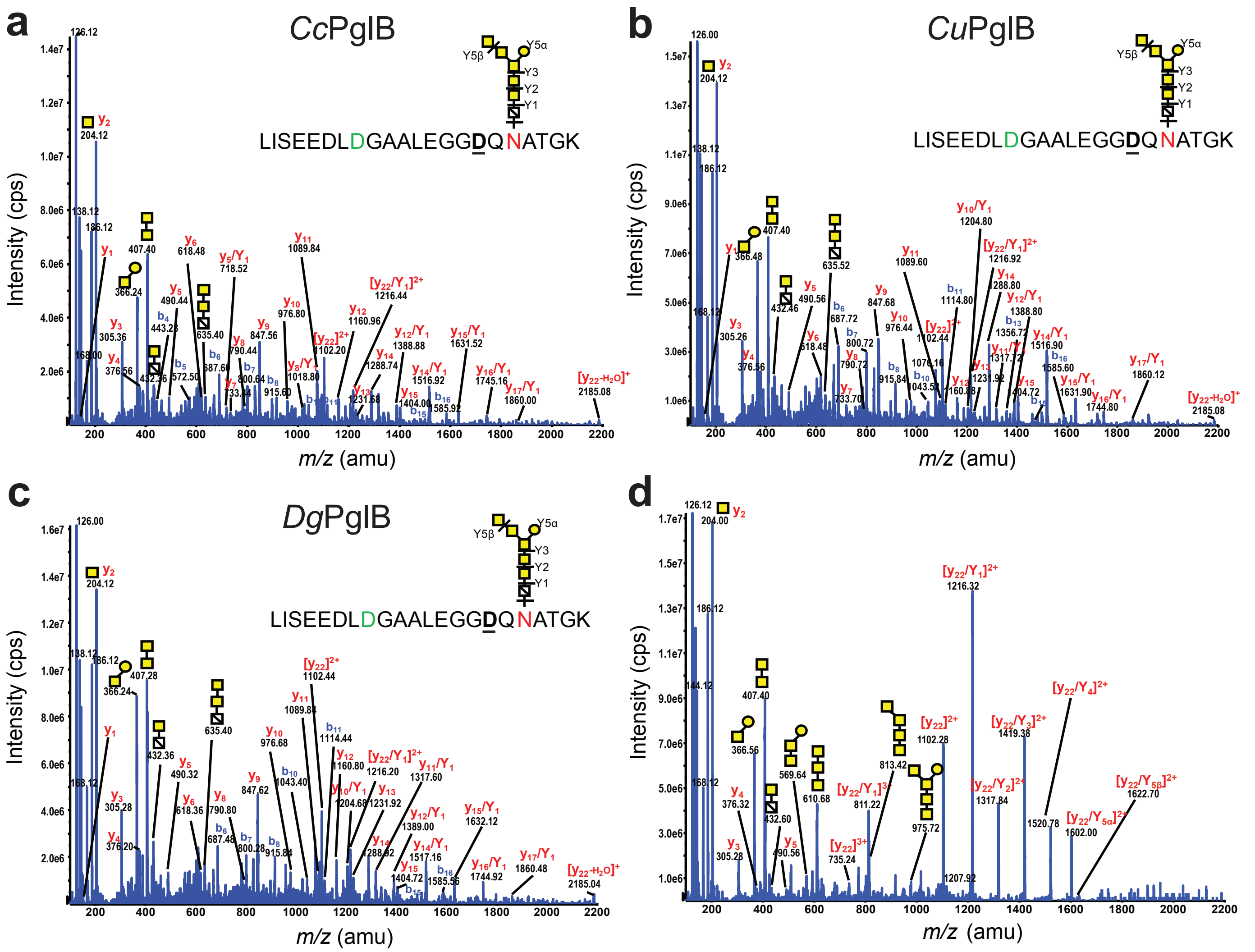

***SI* Figure S3. Mass spectrometry analysis of canonical glycosylation by *Cc*PglB, *Cu*PglB and *Dg*PglB.** MS/MS spectrum of the triply-charged precursor ion for: (b) *Cc*PglB (*m/z* 1203.72); (c) *Cu*PglB (*m/z* 1203.78); and (d) *Dg*PglB (*m/z* 1203.69). These spectra identified the glycopeptide and a 1405.56 Da glycan with bacillosamine as the innermost saccharide attached to the N273 residue (shown in red) in scFv13-R4^{DQNAT}. In each case, a series of y-ions covering from y1 to y15 were observed with the complete knockout of glycan molecule, leading to the confident identification of tryptic peptide: 256- LISEEDLDGAALEGGAQNATGK-277, in which N263 was also found deamidated to Asp, consistent with common deamidation at an Asn residue followed by Gly residue. A second series of y-ions with the added mass of 228.11 Da at N273 was also found covering from y8/Y1 to y17/Y1 for *Cc*PglB and from y10/Y1 to y17/Y1 for *Cu*PglB and *Dv*PglB, providing direct evidence for bacillosamine as the innermost saccharide (Y1) attached to N273. These results are also consistent with the previous observation that a relatively tight bond exists for Y1-peptide compared to the fragile internal glycan bonds. (d) Representative MS/MS spectrum (data from *Dg*PglB is shown) for the quadruply-charged precursor (*m/z* 903.07) with low collision energy (CE = 29 eV) applied. A complete Y-type series of ions (from Y1 to Y5β) attached to the core peptide reveals the expected *C. jejuni* heptasaccharide glycan found in tryptic digest of scFv13-R4^{DQNAT}.

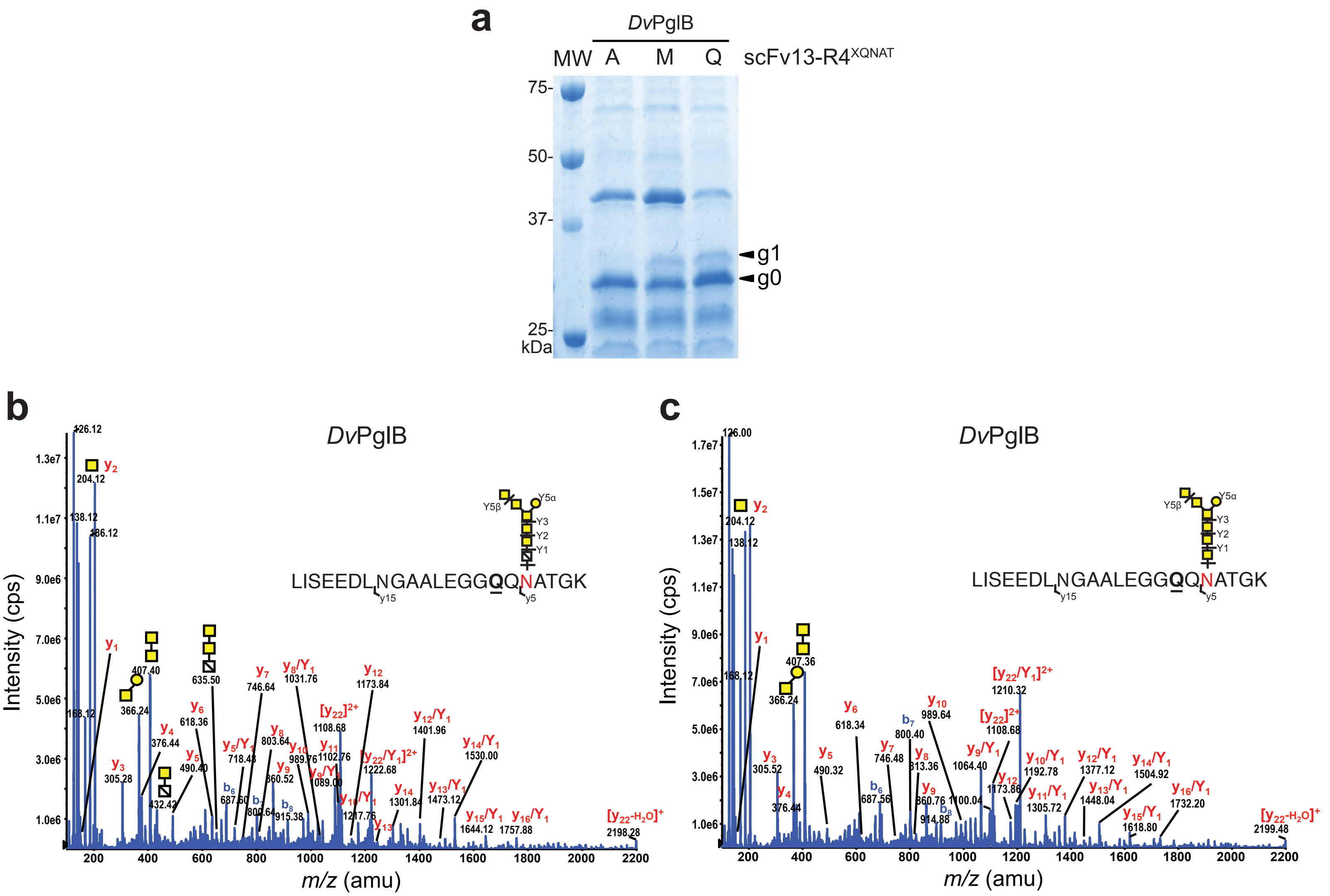

***SI* Figure S4. Mass spectrometry analysis of non-canonical glycosylation by *Dv*PglB.** (a) Ni-NTA-purified scFv13-R4^{AQNAT}, scFv13-R4^{MQNAT}, and scFv13-R4^{QQNAT} samples used in MS analysis, stained with Coomassie Brilliant Blue G-250. Glycosylated bands, indicated by g1 arrow, were excised and submitted for MS analysis. Molecular weight (MW) markers are indicated at left. (b) MS/MS spectrum of the triply-charged precursor (*m/z* 1207.72), identifying the glycopeptide and a 1405.56 Da glycan with bacillosamine as the innermost saccharide attached to the N273 residue (shown in red) in scFv13-R4^{QQNAT}. A series of y-ions covering from y1 to y11 was observed with the complete knockout of glycan molecule, leading to the confident identification of tryptic peptide: 256-LISEEDLNGAALEGGQQNATGK-277. A second series of y-ions with the added mass of 228.11 Da at N273 was also found covering from y8/Y1 to y16/Y1, providing direct evidence for bacillosamine as the innermost saccharide (Y1) attached to N273. (c) MS/MS spectrum on the triply-charged precursor (*m/z* 1199.45), identifying the glycopeptide and a 1380.56 Da glycan with *N*-acetylhexosamine as the innermost saccharide attached to the N273 residue (shown in red) in scFv13-R4^{QQNAT}. A series of y-ions covering from y1 to y12 was observed with the complete knockout of glycan molecule, leading to the confident identification of tryptic peptide: 256-LISEEDLNGAALEGGQQNATGK-277. A second series of y-ions with the added mass of 203.05 Da at N273 was also found covering from y9/Y1 to y16/Y1, providing direct evidence for *N*-acetylhexosamine as the innermost saccharide (Y1) attached to N273. These results are consistent with the previous observation that a relatively tight bond exists for Y1-peptide compared to the fragile internal glycan bonds.

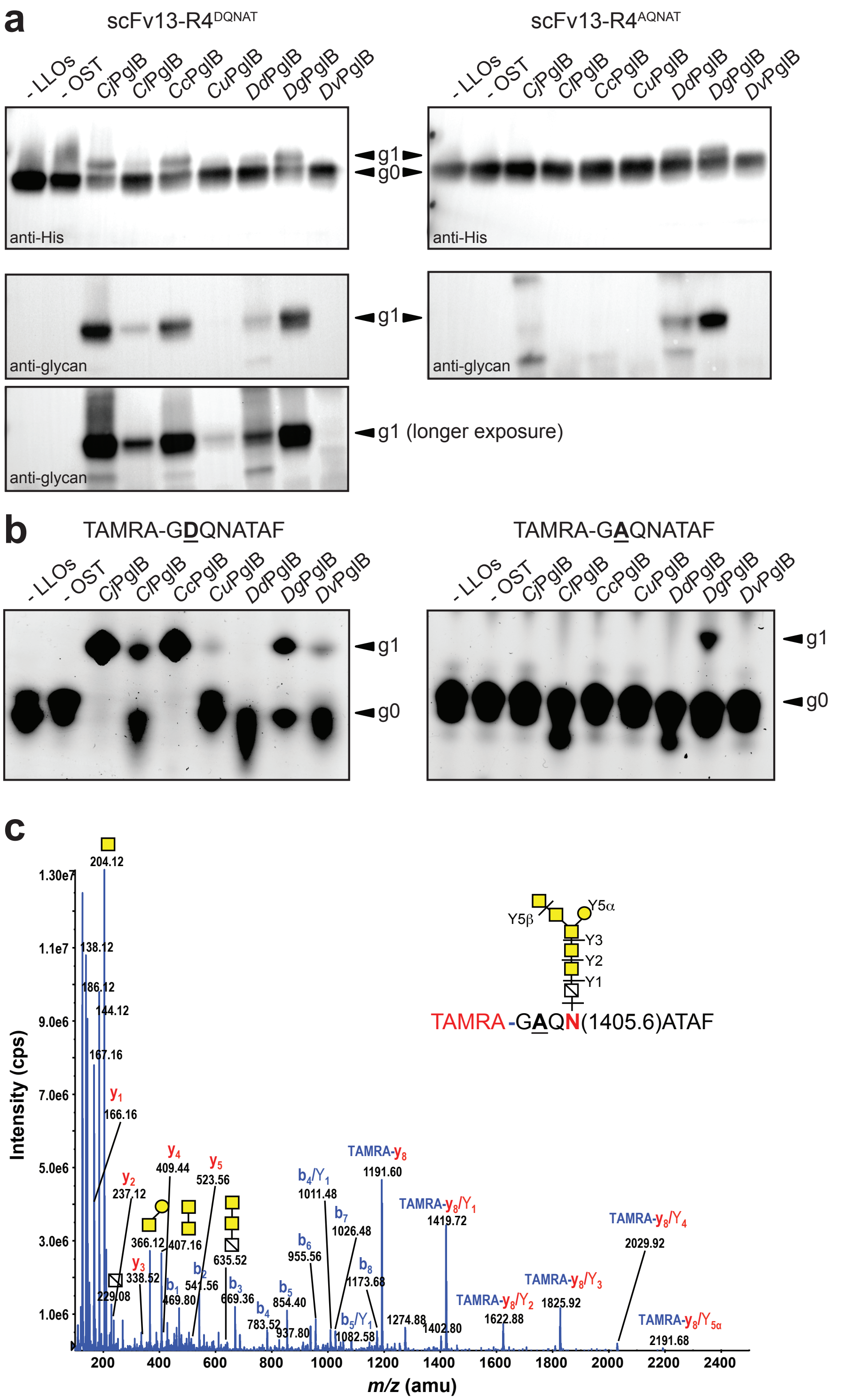

***SI* Figure S5. *In vitro* modification of canonical and non-canonical sequons by PglB homologs.** (a) Immunoblot analysis of *in vitro* glycosylation products generated by incubating purified scFv13-R4^{DQNAT} or scFv13-R4^{AQNAT} with LLOs and one of the PglB homologs indicated. Blots were probed with anti-His antibodies against the C-terminal polyhistidine tag on acceptor protein or anti-glycan hR6 serum reactive with the *C. jejuni* heptasaccharide. The g0 and g1 arrows indicate un- and monoglycosylated acceptor proteins, respectively. Molecular weight (MW) markers are indicated on the left. Panel on bottom left shows longer exposure of the blot in (b) to reveal activity for inefficiently glycosylated targets. (b) Modification of TAMRA-labeled peptides by PglB homologs indicated. Reaction products were separated by Tricine/SDS-PAGE, and fluorescence signals were acquired by an image analyzer. Lanes 1 and 2 are negative controls, where no LLOs (lane 1) or no OST (lane 2) was added to the reaction. The g0 and g1 arrows indicate un- and monoglycosylated acceptor peptides, respectively. Results are representative of two biological replicates. (c) MS/MS spectrum of the triply-charged precursor (*m/z* 866.43), identifying the glycopeptide and a heptascharide glycan (1405.56 Da) with bacillosamine as the innermost saccharide attached to the N4 residue in the TAMRA-modified 8-mer peptide. A series of b-ions covering from b1 to b8, and y-ions were observed with the complete knockout of glycan molecule, leading to the identification of the peptide sequence with TAMRA (412.14 Da) attached to the N-terminal G1 residue. A couple of b-ions (b4 and b5) with the added mass of 228.11 Da at the N4 residue were also detected, providing direct evidence for bacillosamine as the innermost saccharide (Y1) attached to N4. Additional y-type series ions (from Y1 to Y5α) also revealed the expected heptasaccharide glycan structure with bacillosamine as the innermost saccharide linked to the intact peptide.

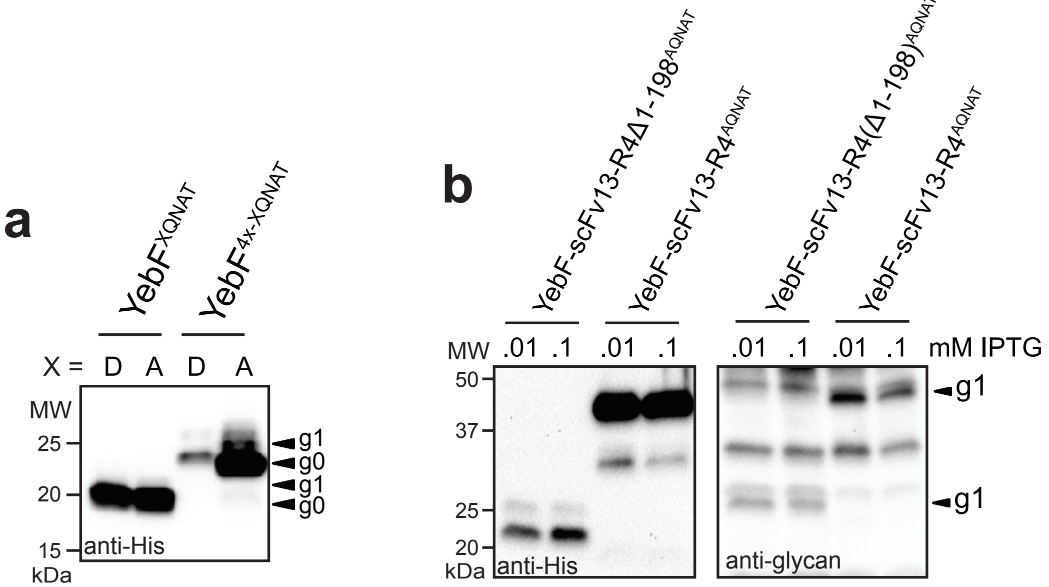

***SI* Figure S6. Adaptation of glycoSNAP assay for screening *Dg*PglB acceptor-site specificity.** (a) Immunoblot analysis of YebF$^{(D/A)QNAT}$ or YebF$^{4x\text{-}(D/A)QNAT}$ glycosylation with the *C. jejuni* heptasaccharide in cells complemented with *Dg*PglB. (b) Immunoblot analysis of YebF-scFv13-R4Δ1-198^{AQNAT} or YebF-scFv13-R4^{AQNAT} glycosylation with the *C. jejuni* heptasaccharide in cells complemented with *Dg*PglB at varying levels of acceptor protein induction. Blots were probed with anti-His antibody or hR6 antiserum containing *C. jejuni* heptasaccharide glycan-reactive polyclonal antibodies. The g0 and g1 arrows indicate un- and monoglycosylated acceptor proteins, respectively. Molecular weight (MW) markers are indicated at left. Results are representative of two biological replicates.

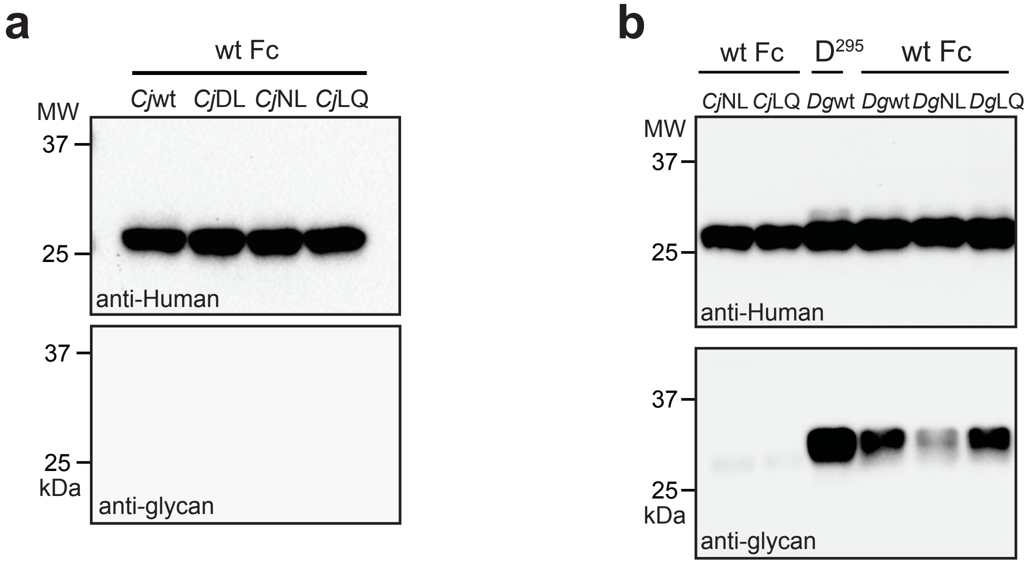

***SI* Figure S7. Unique Fc glycosylation by *Dg*PglB.** Immunoblot analysis of native Fc domain (wt Fc) and Fc^{Q295D} mutant (D^{295}), which places an acidic residue in the -2 position of the native QYNST sequon, co-expressed in the presence of: (a) wild-type *Cj*PglB (*Cj*wt) or previously isolated relaxed acceptor-site specificity variants with DL, NL, or LQ substituted for residues R327 and R328 (*Cj*DL, *Cj*NL, and *Cj*LQ); and (b) *Cj*NL, *Cj*LQ, wild-type *Dg*PglB (*Dg*wt), or *Dg*PglB variants with NL and LQ substitutions in positions R376 and R377 (*Dg*NL and *Dg*LQ, respectively). Molecular weight (MW) markers are indicated to the left. Results are representative of two biological replicates.
